# Supplementary material for: Psychometric evaluation of protective measures in Native STAND: A multi-site cross-sectional study of American Indian Alaska Native high school students
Source: PLoS One. 2022 May 17;17(5):e0268510. doi: 10.1371/journal.pone.0268510 (PMC9113605; doi:10.1371/journal.pone.0268510)
Supplement: S1 File — (DOCX) [file pone.0268510.s001.docx]

S1. Native STAND Strength-based Scales

Social Support (Likert 1=Strongly Disagree-5=Strongly Agree)

1. If I had a personal problem, I could ask someone in my family for help

2. Share thoughts/feelings family

3. I have friends who support me

4. I can talk about my problems with my friends

Community Safety/Community (Likert 1=Strongly Disagree-5=Strongly Agree)

1. I feel safe in my community or neighborhood

2. If I had to move, I would miss the community I now live in

3. I feel safe at home

Self-esteem (Likert 1=Strongly Disagree-5=Strongly Agree)

1. I smile and laugh a lot

2. I adjust well to new situations and challenges

3. I try to do my best

4. I am optimistic about my future

5. I have a sense of what life is calling me to do

6. Sometimes I think I am no good at all (RV)

7. I feel that I am a failure (RV)

Culture (Likert 1=Strongly Disagree-5=Strongly Agree)

1. Being Native American is a major part of my identity

2. I believe that I have many strengths because I am Native American

3. I have spent more time trying to find out more about the history, traditions, and customs of Native people
