# Supplementary material for: Psychometric evaluation of protective measures in Native STAND: A multi-site cross-sectional study of American Indian Alaska Native high school students
Source: PLoS One. 2022 May 17;17(5):e0268510. doi: 10.1371/journal.pone.0268510 (PMC9113605; doi:10.1371/journal.pone.0268510)
Supplement: Attachment — Submitted filename: Response to Reviewers 5-2-22.docx [file pone.0268510.s003.docx]

May 2, 2022

Dear Editor,

We are pleased to resubmit our manuscript for consideration in PLOS One. Our responses to reviewer comments are located in the table below.

Please feel free to contact me if you have additional questions.

Allyson Kelley, DrPH

kelleyallyson@gmail.com

| Reviewer 1 | Response |
| --- | --- |
| Reviewer #1: The authors’ revisions and responses are appreciated. I have two minor suggestions as below.  1. For corrected item-total correlations, the authors could consider to specify which items are the ones with maximum and minimum correlation coefficients in the text. | We appreciate this request and this information was in the paper under Table 2 on pages 7-8 for Self-esteem “Inter-item correlations ranged from 0.213 to 0.329 (average = 0.278) and corrected item-total correlations ranged from 0.361 to 0.628.”, and for Culture, Social support, and Community and Community Safety. This is also a column now in Table 2. Please let us know if you have additional questions. Thank you. |
| 2. In the response to one of the previous comments on self-esteem scale, the authors stated that “Previous research reports that the negative or reverse coded (RV) items were not as reliable as the positively coded items” in Rosenberg's self-esteem scale. Is it one of the reasons why authors excluded three RV items in current version of the scale? If yes, please consider to add this reason and the citation in Method section. | We appreciate this comment and added a citation to the methods section. The self-esteem scale we used included two RV coded items instead of three because of potential reliability issues with RV coded items reported by previous researchers  Marsh, H. W. (1996). Positive and negative global self-esteem: A substantively meaningful distinction or artifactors?. *Journal of personality and social psychology*, *70*(4), 810.  The self-esteem scale was also modeled after the Native VOICES survey. We added appropriate citations in the methods section. |
| Reviewer 2 |  |
| Q1  Line 233, in the part of “discussion”, authors are still using the expression of “AI/AN youth”, which I think may generalize the results of the study. Authors should limit their findings to the sample they investigated, and consistent with the title | We appreciate this feedback. We revised this sentence to be more consistent with the title and indicated results apply to those youth participating in the study. Thanks!  The revised title is, "Psychometric Evaluation of Protective Measures in Native STAND: A Multi-site Cross-Sectional Study of American Indian Alaska Native High School Students" |
| Q2  In the part of “Conclusion”, the authors stated too much about the value of this research for fellow researchers, I understand the necessity of these statements, but in fact, in the “conclusion”, you only need to tell the readers what were the most important finding through the “results” and “discussion” briefly. So, suggestion of research direction for fellow researchers can be moved to the part of "discussion", which I think was too thin.  In conclusion, I have no other comments on this manuscript, if the authors could improve the details, I think it will be valuable research for readers. | We appreciate this feedback and edited the conclusion to highlight the most important finding. We moved the suggestions for future research to the discussion section as well.  Thank you. |
